# Development of a PCR algorithm to detect and characterize *Neisseria meningitidis* carriage isolates in the African meningitis belt

**DOI:** 10.1371/journal.pone.0206453

**Published:** 2018-12-05

**Authors:** Kanny Diallo, Mamadou D. Coulibaly, Lisa S. Rebbetts, Odile B. Harrison, Jay Lucidarme, Kadidja Gamougam, Yenenesh K. Tekletsion, Akalifa Bugri, Aliou Toure, Bassira Issaka, Marietou Dieng, Caroline Trotter, Jean-Marc Collard, Samba O. Sow, Xin Wang, Leonard W. Mayer, Ray Borrow, Brian M. Greenwood, Martin C. J. Maiden, Olivier Manigart, for the MenAfriCar Consortium

**Affiliations:** 1 Centre pour le Développement des Vaccins (CVD), Bamako, Mali; 2 University of Oxford (Department of Zoology), Oxford, United Kingdom; 3 Public Health England, (PHE–Vaccine Evaluation Unit), Manchester, United Kingdom; 4 Centre de Support en Santé Internationale (CSSI), Ndjamena, Chad; 5 Armauer Hansen Research Institute (AHRI), Addis Ababa, Ethiopia; 6 Navrongo Health Research Centre (NHRC), Navrongo, Ghana; 7 Centre de Recherche Médicale et Sanitaire (CERMES), Niamey, Niger; 8 Institut de Recherche pour le Développement (IRD), Dakar, Senegal; 9 University of Cambridge (Disease Dynamics Unit -Department of Veterinary Medicine), Cambridge, United Kingdom; 10 Centers for Disease Control and Prevention, Division of Bacterial Diseases, Atlanta, United States of America; 11 London School of Hygiene and Tropical Medicine (LSHTM), London, United Kingdom; Oklahoma State University, UNITED STATES

## Abstract

Improved methods for the detection and characterization of carried Neisseria meningitidis isolates are needed. We evaluated a multiplex PCR algorithm for the detection of a variety of carriage strains in the meningitis belt. To further improve the sensitivity and specificity of the existing PCR assays, primers for gel-based PCR assays (sodC, H, Z) and primers/probe for real-time quantitative PCR (qPCR) assays (porA, cnl, sodC, H, E, Z) were modified or created using Primer Express software. Optimized multiplex PCR assays were tested on 247 well-characterised carriage isolates from six countries of the African meningitis belt. The PCR algorithm developed enabled the detection of N. meningitidis species using gel-based and real-time multiplex PCR targeting porA, sodC, cnl and characterization of capsule genes through sequential multiplex PCR assays for genogroups (A, W, X, then B, C, Y and finally H, E and Z). Targeting both porA and sodC genes together allowed the detection of meningococci with a sensitivity of 96% and 89% and a specificity of 78% and 67%, for qPCR and gel-based PCR respectively. The sensitivity and specificity ranges for capsular genogrouping of N. meningitidis are 67% - 100% and 98%-100% respectively for gel-based PCR and 90%-100% and 99%-100% for qPCR. We developed a PCR algorithm that allows simple, rapid and systematic detection and characterisation of most major and minor N. meningitidis capsular groups, including uncommon capsular groups (H, E, Z).

## Introduction

*Neisseria meningitidis* has been responsible for numerous outbreaks of septicaemia and meningitis in countries of the African Sahel and sub-Sahel, a region known as the “African meningitis belt”[1]. The devastating nature of this disease, which predominately affects children and young adults, makes its control an international health priority. *N*. *meningitidis* is a common commensal bacterium capable of harmlessly colonizing the upper respiratory tract in a dynamic process referred to as “carriage” [2]. Mucosal carriage is a necessary prerequisite for meningococcal disease. Carriage of *N*. *meningitidis* causes disease when it breaches the host epithelial barriers and invades the bloodstream or penetrates the subarachnoidal cavity and infects the meninges [3].

*N*. *meningitidis* are classified into 12 serogroups (A, B, C, E, H, I, K, L, W, X, Y and Z), based on the structure and chemical composition of their capsular polysaccharide [4]. The serogroups associated with disease in the African meningitis belt are A, W, X, Y and C. With serogroup A epidemics being eliminated following the introduction of the serogroup A conjugate vaccine MenAfriVac[5–7], non-A meningococcal serogroups become the major cause of meningococcal disease in sub Sahelian Africa. Recently, large serogroup C epidemics occurred in Niger and Nigeria, [8] demonstrating the epidemic potential of non- serogroup A meningococci. Timely control and prevention of the epidemics have been a challenge due to vaccine shortages, which has resulted in tremendous pressure on hospital facilities and public health authorities. Serogroup B, a common cause of meningococcal disease in many high-income countries [9], is rarely detected in the meningitis belt. Serogroups E, I, H, K, L, Z are less commonly identified in invasive disease. Queries on the more than 38,633 records on PubMLST.org/*neisseria* website have demonstrated that serogroups E and Z are found both in invasive and carriage specimens, serogroups K, L, H are only found in carriage samples but very rarely, and serogroup I was not found at the time of writing. Furthermore, previous carriage studies in Europe have identified a high proportion of *N*. *meningitidis* isolates, among which, up to 30–45% did not express their capsule genes [10] and 16–20% lacked regions A, B and C of the capsular polysaccharide synthesis (*cps*) genes, known as a capsule null (*cnl*) genotype [11].

Several meningococcal cross-sectional carriage studies have been conducted in the African meningitis belt in recent years, including surveys in Burkina Faso [12, 13], and in seven other countries (Chad, Ethiopia, Ghana, Mali, Niger, Nigeria and Senegal) as part of the MenAfriCar project [14, 15]. Conventional microbiological methods have been used in these studies to detect and characterise meningococci, but these methods are time consuming, expensive and susceptible to errors. Consequently, many African laboratories have been increasingly adopting molecular biology tools to detect and characterise *N*. *meningitidis* and its capsular genotypes as they are more sensitive, specific, and less time consuming than bacteriological methods. DNA-based methods such as Polymerase Chain Reaction (PCR) have greatly improved laboratory diagnosis [16]; many studies have developed reliable PCR assays for the detection of *N*. *meningitidis* [17–19] and characterization of its capsular genogroup using specific genes. However, most studies have focused on the common disease serogroups, A, B, C, W, X and Y [20–23]. Fewer studies have investigated PCR for the detection of *cnl* or serogroups E, H, and Z which are mostly found in carriage [20, 24]. Some research groups have developed gel-based PCR assays for the detection and genogrouping of *N*. *meningitidis* whereas others have used real-time quantitative PCR (qPCR) protocols. The choice of one technique over another is often dictated by budgetary considerations, as qPCR is more expensive but less subject to contamination. Evaluation of the impact of meningococcal conjugate vaccines requires sensitive and specific PCR assays that cover all serogroups to accurately and thoroughly assess the epidemiology of *N*. *meningitidis* carriage and disease, including the potential emergence of less common groups through strain replacement and/or capsule switching [25].

The present study was conducted within the MenAfriCar project, which collected pharyngeal swabs from over 60,000 subjects in seven countries within the African meningitis belt [15]. Our objectives were to improve *N*. *meningitidis* carriage assessment by developing and optimising a simple, accurate and standardised multiplex PCR algorithm for the detection of *N*. *meningitidis* species and its capsular genogroups including: (i) the most prevalent genogroups A, B, C, X, Y and W (ii) carriage-associated genogroups such as E, H, and Z; and (iii) unencapsulated capsule null meningococci. Both traditional gel-based PCR and qPCR that can be adapted to African laboratories were validated using carriage isolates collected as part of MenAfriCar studies.

## Material and methods

### Literature search for available primers/probes

A literature search was undertaken to identify PCR methods with primers and probes published on *N*. *meningitidis* detection and characterization. The NCBI PubMed database was used with search terms “*Neisseria meningitidis”* and “PCR” (date of access: November 2011). The different techniques and detection algorithms were evaluated in order to define the most appropriate ones for our context. A focus was placed on summarising the best gel-based and qPCR assays already existing for detection and characterization of meningococci.

### In silico specificity screen—Evaluation of selected primers and probes

A total of 112 primers and 16 probes reported in the literature were chosen for further analysis. Firstly, nucleotide mismatches were identified through the comparison with consensus sequences derived from target genes obtained from the pubMLST.org/neisseria website (accessed on September 2012). Primer and probe sequences were compared to all available allelic variants of the target genes to determine whether they were conserved in the majority of alleles available at that time. Technical checks on all primers and probes were then undertaken using the *Primer Express* software design guidelines (Applied Biosystem International (ABI), Villon sur Yvette, France): melting point temperatures (Tm) and potential secondary structures were assessed with optimal Tm varying between 58–60°C and 68–70°C for primers and probes respectively. Finally, nucleotide BLAST searches were undertaken using the NCBI BLAST tool to identify possible cross-reactions with other bacteria [26]. Primers and probes with more than 3 base pair (bp) differences compared to the consensus sequences were not used. Secondary structures or self-dimers were minimised, to avoid their potential detrimental effect on the efficiency of the reaction, but their presence was not a systematic criterion for rejection. Sequences were retained if they displayed 100% coverage according to the NCBI BLAST tool. When the result of these evaluations was unsatisfactory, new primers and/or probes were created.

### Positive controls

Positive controls consisted of purified DNA from genogroups: A (isolate name: Z2491); W (A22); X (860060); Y (71/94); B (EG329, BZ198); C (L93/4286); Z (0084/93); E (297–0); H (H/ASH/87); and capsule null (0083/93) [27] as well as two genogroup E isolates (M07746, M22445). Reference strains for genogroups A and X, were purchased from the American Type Culture Collection (ATCC). Another confirmed capsule null isolate from the first Malian MenAfriCar cross-sectional study (sample 01-010664-XS1) (18) was used as a *cnl* positive control.

### Carriage isolates

*N*. *meningitidis* isolates were collected during the first MenAfriCar cross-sectional studies as previously described [15]. Briefly, pharyngeal swabs were obtained from participants in the rural and urban sites of six centres in Chad, Ethiopia, Ghana, Mali, Niger and Senegal. Suspected *N*. *meningitidis* were subjected to oxidase test, Gram stain, biochemical tests and sero-agglutination [28]. Boiled cell suspensions to release high concentration of DNA were prepared in each center as part of the MenAfriCar routine protocol from bacteria grown overnight on blood agar plates [15]. Extracted DNA samples from all oxidase positive bacteria were also sent to the University of Oxford [14] for molecular characterization. A subset of characterized samples was chosen to constitute a representative panel of the most prevalent *N*. *meningitidis* genogroups as well as non *N*. *meningitidis* from each country. Next, the selected boiled cell suspension extracted DNA samples were sent to the Center for Vaccine Development in Mali (CVD-Mali) where PCR validation was conducted. A total of 247 extracted DNA samples were received, with 42, 38, 18, 46, 44 and 59 samples from Chad, Ethiopia, Ghana, Niger, Mali and Senegal, respectively.

### Characterisation of N. meningitidis at the University of Oxford, UK

*Neisseria* species were analysed using the *rplF* assay [29]. All samples were also tested with a capsule null (*cnl*) sequencing assay [11] or sequencing of a fragment of the *ctrA* gene allowing the characterisation of genogroup A, E, K, L, X and Z. Samples that were positive for *N*. *meningitidis* and negative for *cnl* were tested by a qPCR multiplex assay to determine genogroups A, B, C, W, X and Y [23]. These results were considered as the reference or gold standard for this study since the assays described above were already validated and published.

### Novel gel-based PCR assay design and optimization in Bamako, Mali

New primers were designed for *sodC*, genogroup Z, and H using the online tools Primer 3 and Primer quest (Table 1)[30, 31]. A ready-to-load Master Mix (rlMMx) from Solis Biodyne, stable at room temperature and therefore very convenient for transport and testing in Africa, was employed. rlMMx with different concentrations of MgCl_2_ were tested to find the optimal concentration for the assay. Thermal cycling conditions were the same for all tests: 1 cycle of 3 minutes (min) at 95°C; 35 cycles of 30 seconds (s) at 95°C; 30 s at 55°C; 30 s at 72°C. This was followed by 1 cycle of 10 min at 72°C and a final conservation step at 4°C.

**Table 1:** Primer sequences for gel-based PCR.

Gene	Primer name	Sequence	Start/end	Size	Reference
*porA*	*porA* 2F	GCG GTT TTG CCG GGA ACT AT	755–1017	251	Bennett, DE, 2006
	*porA* 15R	AGT GGC GGC AAT TTC GGT CGT ACT			
*galE-cnl-tex*	GH26R (cnl aF)	GGT CGT CTG AAA GCT TGC CTT GCT C	250–226	432	Claus, H, 2002
	HC344 (cnl aR)	GGA TTG GAC GAG CGA GAC	72–55		
*sodC*	*sodC*-F2	GCG GTT AGT GCA GTA TGT TCA G	113–641	530	Present study
	sodC-R2	TAA TCA CGC CAC ATG CCA TA			
*mynB/csaB*	98–28 (AF)	CGC AAT AGG TGT ATA TAT TCT TCC	270–664	395	Taha, MK, 2000
	98–29 (AR)	CGT AAT AGT TTC GTA TGC CTT CTT			
*siaD (csw)*	98–32 (W135F)	CAG AAA GTG AGG GAT TTC CAT A	911–1030	120	Taha, MK, 2000
	98–33 (W135R)	CAC AAC CAT TTT CAT TAT AGT TAC TGT			
*Xcba (csxA)*	X-10 (F)	ACA GCC CAT AAA ACA CCC GTA TCA TC	142–343	202	Yaro, S, 2012
	X-11 (R)	GTG ATT GGA ATC TTC CAA TAT CGG T			
*siaD (csb)*	98–20 (BF)	GCA TGC TGG AGG AAT AAG CAT TAA	645–1099	455	Taha, MK, 2000
	98–19 (BR)	GGA TCA TTT CAG TGT TTT CCA CCA			
*siaD (csc)*	98–17 (CF)	TCA AAT GAG TTT GCG AAT AGA AGG T	789–1054	266	Taha, MK, 2000
	98–18 (CR)	CAA TCA CGA TTT GCC CAA TTG AC			
*siaD (csy)*	98–34 (YF)	CTC AAA GCG AAG GCT TTG GTT A	911–1030	120	Taha, MK, 2000
	98–35 (YR)	CTG AAG CGT TTT CAT TAT AAT TGC TAA			
*cshC*	cshC-F1	GTG CCG ATA TTG CCT CAG AT	2378–2533	156	Present study
	cshC-R1	CTT CGG ATG GGA ACT TGA AA			
*cap29EH*	39429 (29E aF)	TTG GCG GTT GAA ACC TTA C	22–715	694	Zhu, H, 2011
	39430 (29E aR)	GCG TAT CAT GCT CCA TTA CCA			
*cszC*	cszC-Fwd (ZF)	AGG TTC ATC TGC TGG GAT TAC GCT	3506–3806	301	Present study
	cszC-Rev (ZR)	AAG CGA TTA ATG GCC TGT TGC TGG			

Sequences, positions and amplicons size of each primer pair used in the 4 multiplexes. In bold are base pairs that showed a mismatch with at least one of the alleles present in pubMLST, but not with the majority of them.

### Novel quantitative real-time PCR (qPCR) assay design and optimization in Bamako, Mali

Although our assay was developed to determine whether a specimen is positive or negative for meningococci and their genogroups, we keep the term “qPCR” to comply with the MIQE guidelines [32]. Quantification of meningococci would not add any information in our context since we were working with DNA samples extracted from bacterial culture. Primers and probes were designed for the *N*. *meningitidis* species-specific gene *porA* and for the non-species-specific *cnl*, and a modified species-specific *sodC* probe was designed based on a previously published method [18]. Genogroups were determined using the 2^nd^ (A, W, X) and 3^rd^ (B, C, Y) qPCR multiplexes developed by Wang *et al*. [23]. Primers and probes for these genogroups were kindly provided by the Centers for Disease Control and Prevention (CDC). Primers and probes were also designed for genogroups E, H, and Z using the Primer Express software from ABI. Minor Groove Binding (MGB) hydrolysis probes were designed, as opposed to Black Hole Quencher (BHQ) hydrolysis probes which are more difficult to manufacture. The *sodC* probe was modified in Primer Express to make it 12 base pairs shorter and accommodate an MGB quencher (Table 2). All primers/probe were tested at the following concentrations in a 25 μl solution: 900nM/250nM; 600nM/200nM; 300nM/100nM and 100nM/50nM respectively. Optimal concentrations in a TaqMan Gene Expression MasterMix (reference: 4369016 from ABI) were identified for each primers/probe set and used subsequently in the different assays. Cycling conditions were similar for all tests: 1 cycle of 2 min at 50°C, 1 cycle of 10 min at 95°C followed by 50 cycles of 15 s at 95°C and 1 min at 60°C. The ABI 7500 fast cycler was used to perform the reaction and the results analysed using the 7500 Fast software (see dx.doi.org/10.17504/protocols.io.rjsd4ne for detailed information).

**Table 2:** Primer and probe sequences for qPCR.

Gene	Primer/Probe name	Sequence	Start/end	Concentration (nM)	Reference
*porA*	*porA_fwd_1*	GCC GGC GTT GAT TAT GAT TT	1126–1145	600	Present study
	*porA_rev_1*	AGT TG**C** CGA T**G**C **C**GG TAT T	1210–1192	600	
	*porA_Pb_1* FAM (MGB)	CTT CCG CCA TCG TGT **C**	1157–1172	200	
*sodC*	Nm *sodC* FWd 351	GCA CAC TTA GGT GAT TTA CCT GCA T	446–470	600	Dolan, T, 2011
	Nm sodC Rev 478	CCA CCC GTG TGG ATC ATA ATA GA	551–573	600	
	*sodC* Pb387 NED (MGB)	CAT GAT GGC ACA GCA A	482–497	100	Present study
*galE/cnl/tex*	cnl_fwd_2	GAA TT**G** CAT AGG TTA TC**C** AAA AT**C** AC	110–85	900	Present study
	cnl_fwd_2b	GAG TT**G** CAC AGA TTA TC**C** AGA AT**C** AC	110–85	900	
	cnl_rev_4	TTT GCC CGA TAC AAT CTG AAA G	85–106	900	
	cnl_Pb_2 VIC (MGB)	ATA AAA CCG GTG CCG CC	38–22	250	
*csw/synG*	F857	TAT TTA TGG AAG GCA TGG TGT ATG	935–958	600	Wang, X, 2011
	R964	TTG CCA TTC CAG AAA TAT CAC C	1063–1042	600	
	Pb907i FAM (BHQ)	AAA TAT GGA GCG AAT GAT TAC AGT AAC T**A**T AAT GAA	985–1020	200	
*csaB/sacB*	F2531	AAA ATT CAA TGG GTA TAT CAC GAA GA	934–959	900	Mothershed, EA, 2004
	R2624	ATA TGG TGC AAG CTG GTT TCA ATA G	1025–1001	900	
	Pb2591i HEX (BHQ)	CTA AAA GTA GGA AGG GCA CTT TGT GGC ATA AT	992–961	250	
*csxB/xcbB*	F173	TGT CCC CAA **C**CG TTT ATT GG	429–448	900	Mothershed, EA, 2004
	R237	TGC TGC TAT CAT AGC CGC C	493–475	900	
	Pb196 CY5 (BHQ)	TGT TTG CCC ACA TGA ATG GCG G	452–473	250	
*csb/synD*	F737	GCT ACC CC**A** TTT CAG ATG ATT TGT	737–760	900	Wang, X, 2011
	R882	ACC AGC CGA GGG TTT ATT TCT AC	905–883	900	
	Pb839i CY5 (BHQ)	AAG AGA T**G**G GYA ACA ACT ATG TAA TGT CTT TAT TT	839–873	250	
*csc/synE*	F478	CCC TGA GTA TGC GAA AAA AAT T	591–612	600	Mothershed, EA, 2004
	R551	TGC TAA TCC CGC CTG AAT G	664–646	600	
	Pb495i FAM (BHQ)	TTT CAA TGC TAA TGA ATA CCA CCG TTT TTT TGC	612–644	200	
*csy/synF*	F787	TCC GAG **C**AG GAA ATT TAT GAG AAT AC	865–890	900	Wang, X, 2011
	R929	TTG CTA AAA TCA TTC GCT CCA TAT	1010–987	900	
	Pb1099i HEX (BHQ)	TAT GGT GTA CGA TAT CCC TAT CCT TGC CTA TAA T	948–981	250	
*cseE/cap29e*F	cseE-2_Fwd1	GAGGCTGGCAATGACCAATT	470–488	600	Present study
	cseE-2_Rev1	CCCAGCATATCGACAACCAA	548–529	600	
	cseE-2_Pb1 FAM (MGB)	ATCTTATGTGAACGTGGCGC	490–509	200	
cszC/capZD	cszC-1_Fwd1	CAG GCC GAA GAG CGT TAT CA	895–914	900	Present study
	cszC-1_Rev1	CGC CAT TCA GGG CGA TT	950–934	900	
	cszC-1_Pb1 NED (MGB)	ACA GCT CTG GCC TTA G	916–931	250	
cshC	cshC-1_Fwd1	AAG CCC GTT CCA AGA TCA TG	1491–1510	600	Present study
	cshC-1_Rev1	GCG GTT TGG AGA AAT AAT ATG TGT T	1563–1539	600	
	cshC-1_Pb1 VIC (MGB)	AAT GTC AGC CGT AAC TT	1513–1529	200	

Sequences, positions and concentrations of primers and probes used in the 4 multiplexes, MGB: Minor Groove Binding/ BHQ: Black Whole Quencher, are two different types of Quencher. The probes obtained from CDC had a BHQ while the ones designed for this study the MGB quencher. In bold are base pairs that showed a mismatch with at least one of the alleles present in pubMLST, but not with the majority of them.

### Study design and statistical analyses

**Fig 1:**
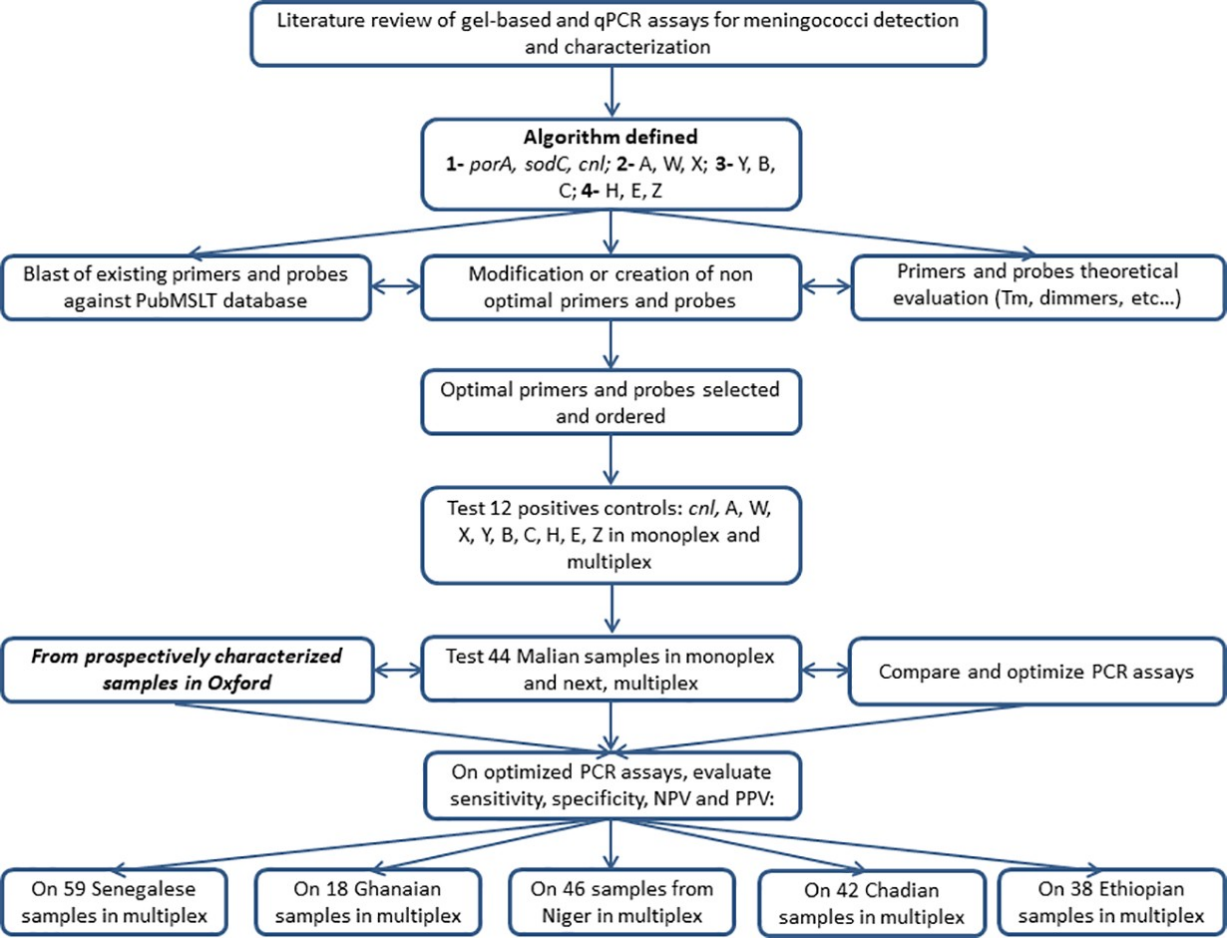
Summary of the methodology employed in the study and samples tested.

Initially, 16 positive controls, representing all target genogroups (A, B, C, W, X, Y, H, E, Z, *cnl*), were used for the optimization of both gel-based and qPCR in monoplex and appropriate multiplexes. Optimal qualitative results from monoplex and multiplex assays were evaluated for gel-based PCR by visualizing the quality and strength of the bands, whereas optimal conditions were defined for the qPCR assay using quantitative Ct values. Then, 44 Malian isolates were analysed using all primers and probes in monoplex and multiplex assays in parallel to further evaluate the optimized PCR conditions for multiplex assays and ensure the multiplex assays were comparable to monoplex assays. Later, as more samples were prospectively characterized at Oxford University from other MenAfriCar countries, samples from Chad, Ethiopia, Ghana, Niger and Senegal were tested with the optimized multiplex assays (Fig 1). Sensitivity, specificity, positive predictive value (PPV) and negative predictive value (NPV) (see definitions in Table 3) were calculated for primers/probe triplets (forward primer, reverse primer and probe for each qPCR) using the results obtained from Oxford University as a gold standard.

**Table 3:** Definition of sensitivity, specificity, positive predictive value and negative predictive value.

Carriage of meningococci (confirmed by the gold standard in Oxford):		
PCR outcome/carriage	Carriage present	No carriage
Positive	True Positive (TP)	False Positive (FP)
Negative	False Negative (FN)	True Negative (TN)

Sensitivity (SS): Proportion of samples positive by the gold standard technique (true carriers) that were positive by PCR. Sensitivity = TP/(TP+FN)

Specificity (SP): Proportion of samples negative by the gold standard technique (true non-carriers) that were negative by PCR. Specificity = TN/(TN+FP)

Positive Predictive Value (PPV): Probability to have a positive result with the new PCR when it is positive with the gold standard (carriage of meningococci or carriage of the tested strain). PPV = TP/(TP+FP)

Negative Predictive Value (NPV): Probability to have a negative result with the new PCR when it is negative with the gold standard (no meningococcal carriage or carriage of the tested strain). NPV = TN/(TN+FN)

## Results

### Literature search and evaluation of primers and probes

Eleven papers describing 112 primers and 16 probes for gel-based or qPCR methods to detect and characterize meningococci were identified (S1 Table). Only papers that described the primer and probe sequences were used. Gel-based PCR assays targeting different genes were identified for *N*. *meningitidis* detection: 1 assay for *crgA*, 2 for *porA*, 2 for *ctrA* and 1 for *sodC*. Similarly, qPCR assays for characterization of genogroups were analyzed: 3 for A, 3 for W, 7 for X, 5 for B, 4 for C, 3 for Y, 1 for H, 2 for E, and 1 for Z.

For the gel-based PCR assays: the same primers were often chosen for detection and genogrouping in different papers. The most frequent were for characterization of genogroups A, W, Y, B, C [22], and X [33]. For the qPCR assays: primers/probe triplets (forward primer, reverse primer and probe) were retrieved for *porA* (1 triplet), *ctrA* (2 triplets), and *sodC* (1 triplet) for the detection of *N*. *meningitidis* and for genogroups A (1 triplet), W (2 triplets), X (1 triplet), B (3 triplets), C (3 triplets), Y (2 triplets). In one of the most recent comprehensive assays, two multiplex PCR for genogroups A, W, X and B, C, Y were used successively [23].

Nineteen (14 primers and 5 probes–see Tables 1 and 2) out of the 68 primers and probes sequences evaluated within the PubMLST database had 1 to 3 mismatches, compared with the consensus sequences, which was considered acceptable. The Tm and dimer analyses generally gave acceptable results for gel-based and qPCR, except for H and Z that had to be redesigned to fit the cycling conditions in the gel-based PCR assay (Tables A, B, C, D, E, F, G and H in S2 Table). A final list of 47 primers and 12 probes was evaluated for our detection and characterization of the PCR algorithm (Tables 1 and 2). Primers and probes regions are represented schematically on their respective loci in Panels A, B, C and D in S1 Fig.

### Optimisation of the four multiplex assays (1- porA, sodC, cnl; 2- A, W, X; 3- Y, B, C; 4- H, E, Z)

Sixteen positive controls from different genogroups were used to optimize both the gel-based and the real-time PCR assays.

For gel-based assay, the optimal MgCl2 concentration for the assay was determined to be 10.4mM and this concentration was retained for the remaining experiments. Each reaction consisted of a 30μl solution with 0.5μl of each primer, 4μl of rlMMx (monoplex) or 6μl of rlMMx (multiplex) and molecular biology grade water (ddH2O).

Table 2 presents the optimal concentrations for the selected primers and probes for real-time PCR (qPCR). The qPCR reaction consisted of a 25μl solution with 12.5 μl of 2X Gene Expression Master Mix and ddH_2_O.

#### Evaluation of sensitivity, specificity, PPV and NPV compared with our gold standard (S3 Table)

**Gel-based assay (Table A in S3 Table)**:

Sixteen positive controls and the samples from Mali (n = 44) were used to compare monoplex and multiplex PCR. All meningococci identified at the University of Oxford were correctly detected and characterized by monoplex gel-based PCR resulting in a sensitivity of 100% for all genes except 75% for genogroup Y (see S3 Table). The *porA* and *sodC* sensitivities were decreased to 82.14% and 96.43% respectively when working in multiplex whereas it was 100% for *cnl* and all genogroups. The specificity of *porA* assay increased from 77.78% in monoplex to 88.89% in multiplex, whereas specificity of *sodC* decreased from 29.64% in monoplex to 25.93% in multiplex. Specificity was 100% for *cnl* and all genogroups except X and Z with 96.55% and 96.30% respectively in monoplex and 100% for *cnl* and all genogroups except X and B with 96.55% and 96.15% respectively in multiplex (Supplemental Table 3).

**Fig 2:**
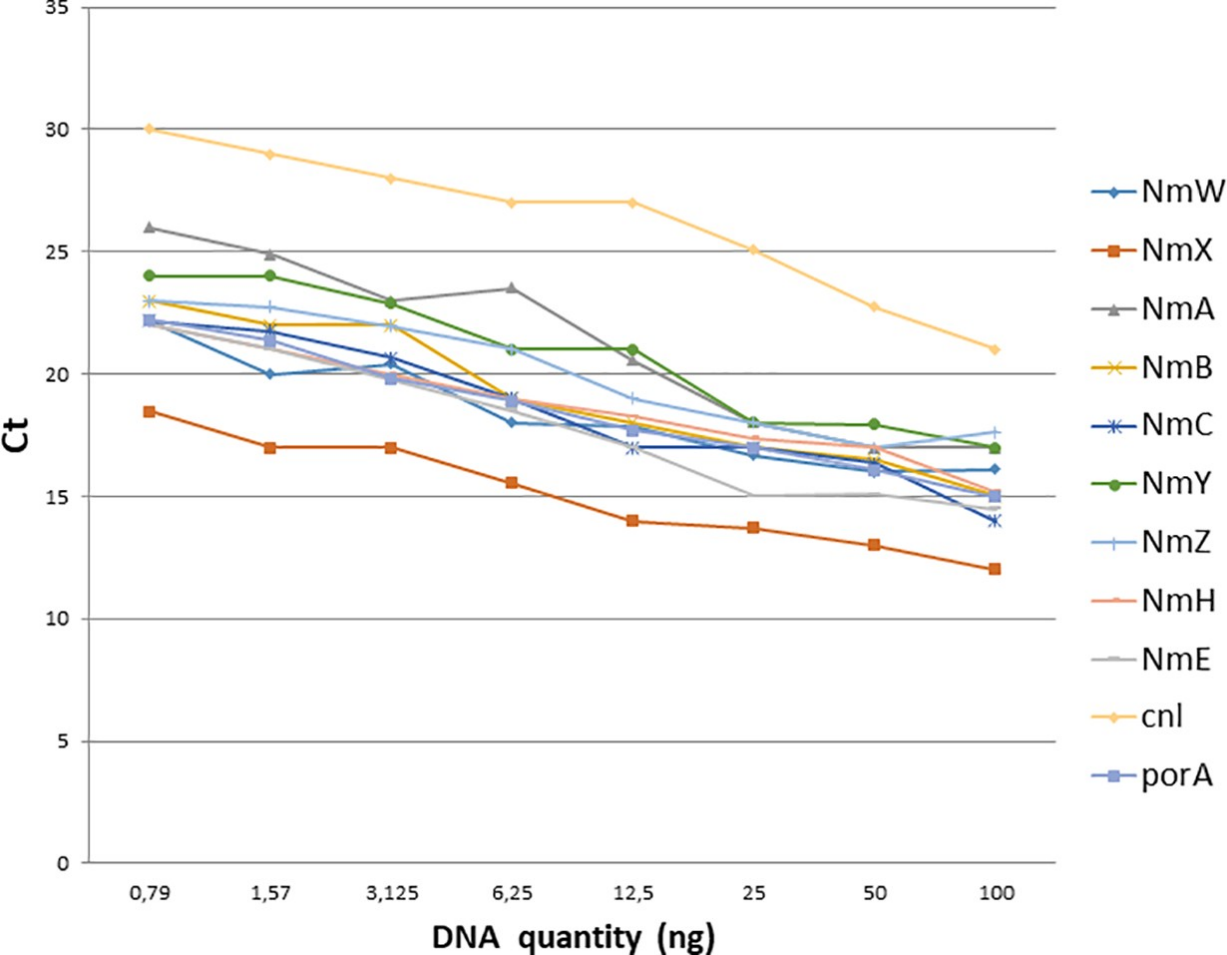
qPCR standard curves. Eight two-fold dilutions of DNA from each positive control were performed and tested in a qPCR assay with appropriate primers and probes. The Ct observed for each dilution is plotted against the DNA concentration demonstrating a decrease in Ct with increased DNA concentration and detection for all genogroups with a threshold of 30. *sodC* was empirically tested later and demonstrated the same threshold (data not shown).

**Real-time PCR assay (Table B in S3 Table)**:

Starting with 100ng of DNA, eight 2-fold serial dilutions were performed to determine the Ct limit for categorizing a sample as positive. The last dilution, at a concentration of 0.79ng, systematically had a Ct lower or equal to 30. This is roughly equivalent to 40,000 genome copies which is far lower than the expected number of colony forming units after culture. The Ct limit was determined to be 30 cycles for crude DNA isolates. For this assay, a sample with a Ct lower or equal to 30 was considered positive and samples above 30 were considered negative (Fig 2).

The sensitivity was 100% for all PCR assays evaluated in this study both in monoplex and multiplex. Specificity was poor for *sodC* with 25.93% in monoplex and 29.63% in multiplex. Specificity for *porA* was slightly decreased from 81.48% to 74.07% in multiplex. All *cnl* and genogroups had a specificity of 100% both in monoplex and multiplex except genogroup X with 96.55% in monoplex. No statistical differences were observed between the new MGB *sodC* probe and the original BHQ *sodC* probe; however, the new MGB probe detected *N*. *meningitidis* at lower Ct values (data not shown) and was included in the final assay.

### Evaluation of the optimized multiplex assay using the pool of African carriage isolates (Table 4)

The best performing assay was tested in multiplex on 203 isolates from five other countries of the MenAfriCar study with the gel-based and qPCR assays making a total of 247 samples. Again, the comparison was made with the gold standard results from Oxford: for the detection by gel-based PCR assay, the best sensitivity was obtained with *sodC* (80.92%—with a specificity of 69.47%) and the best specificity with *porA* (93.68% with a sensitivity of 70.39%). Sensitivity for *cnl* and all genogroups ranged from 66.67% (genogroup X) to 100% for genogroup B and E, whereas specificity ranged from 97.99% (*genogroup X*) and 100% (genogroups C, Y, H and Z).

For the qPCR assay, the best sensitivity and specificity was obtained with *porA* (96.05% and 91.58% respectively) showing no advantage of using *sodC* (94.74% and 77.89% respectively). For *cnl* and all genogroups, sensitivity ranged between 90% (genogroup A and Y) and 100%, whereas specificity ranged between 99.33% (genogroup X) and 100% (Table 4).

**Table 4:** Statistical analysis results for the multiplex gel-based and qPCR assays.

Gel-based								
multiplex								
	SS	SS 95% CI	SP	SP 95% CI	PPV	PPV 95% CI	NPV	NPV 95% CI
porA	70.4	62.4, 77.5	93.7	86.8, 97.6	94.7	88.8, 98.0	66.4	57.8, 74.3
sodC	80.9	73.7, 86.8	69.5	59.2, 78.5	80.9	73.8, 86.8	69.5	59.2, 78.5
Cnl	85.5	75.6, 92.5	98.7	92.9, 100	98.5	91.8, 100	87.2	78.3, 93.4
A	75.0	50.9, 91.3	99.2	95.9, 100	93.8	69.8, 99.8	96.3	91.6, 98.8
W	81.5	61.9, 93.7	98.4	94.3. 99.8	91.7	73.0, 99.0	96.1	91.1, 98.7
X	66.7	9.4, 99.1	98.0	94.2, 99.6	40.0	5.3, 85.3	99.3	96.3, 100
B	100.0	29.2, 100	99.3	96.3, 100	75.0	19.4, 99.4	100.0	97.5, 100
C	80.0	28.3, 99.5	100.0	97.5, 100	100.0	39.8, 100	99.3	96.3, 100
Y	80.0	44.4, 97.5	100.0	97.4, 100	100.0	63.1, 100	98.6	95.1, 99.8
H	NA	NA	100.0	97.6, 100	NA	NA	100.0	97.6, 100
E	100.0	2.5, 100	99.3	96.3, 100	50.0	1.3, 98.7	100.0	97.6, 100
Z	NA	NA	100.0	97.6, 100	NA	NA	100.0	97.6, 100
porA/sodC	88.8	82.7, 93.3	67.4	57.0, 76.6	81.3	74.6, 86.9	79.0	68.5, 87.3
Real-time								
multiplex								
	SS	SS 95% CI	SP	SP 95% CI	PPV	PPV 95% CI	NPV	NPV 95% CI
porA	96.1	91.6, 98.5	91.6	84.1, 96.3	94.8	90.0, 97.7	93.5	86.5, 97.6
sodC	94.7	89.9, 97.7	77.9	68.2, 85.8	87.3	81.2, 91.9	90.2	81.7, 95.7
Cnl	94.7	87.1, 98.5	100.0	95.3, 100	100.0	95.0, 100	95.0	87.7, 98.6
A	90.0	68.3, 98.8	100.0	97.2, 100	100.0	81.5, 100	98.5	94.7, 99.8
W	92.6	75.7, 99.1	100.0	97.1, 100	100.0	86.3, 100	98.4	94.4, 99.8
X	100.0	29.2, 100	99.3	96.3, 100	75.0	19.4, 99.4	100.0	97.5, 100
B	100.0	29.2, 100	100.0	97.5, 100	100.0	29.2, 100	100.0	97.6, 100
C	100.0	47.8, 100	100.0	97.5, 100	100.0	47.8, 100	100.0	97.5, 100
Y	90.0	55.5, 99.7	100.0	97.4, 100	100.0	66.4, 100	99.3	96.2, 100
H	NA	NA	100.0	97.6, 100	NA	NA	100.0	97.6, 100
E	100.0	2.5, 100	99.3	96.4, 100	50.0	1.3, 98.7	100.0	97.6, 100
Z	NA	NA	100.0	97.6, 100	NA	NA	100.0	97.6, 100
porA/sodC	96.1	91.6, 98.5	77.9	68.2, 85.8	87.4	81.4, 92.0	92.5	84.4, 97.2

Results of both PCR assays tested only in multiplex on a panel of 247 samples from Chad, Ethiopia, Ghana, Mali, Niger and Senegal. Sensitivity, Specificity, PPV and NPV were assessed for each primers pair and primers/probe in the multiplex assays and presented as percentages (%) with 95% confidence intervals (CI).

The benefit of evaluating results from *sodC* and *porA* together, instead of individually from the same multiplex, was analysed by comparing each primers/probe sets taken individually or together. Sensitivity and NPV were improved by using both genes together and considering positive any samples that were positive for at least one of the targets for the gel-based PCR assay in comparison with *porA* or *sodC* alone from the same multiplex whereas the specificity and PPV were decreased. For the qPCR assay all statistical results for *sodC* were lower or equal to those for *porA*. Inclusion of *sodC* did not provide additional value (Table 4).

From the panel of 247 samples from all six different countries, 152 were characterized as *N*. *meningitidis* at the University of Oxford using *rplF* sequencing [29]. Of these, 88.82% (135) isolates were correctly identified as meningococci by the gel-based PCR assay and 96.05% (146) by the qPCR assay. All genogroups, but H and Z, were identified in the panel tested: the most commonly represented was the *cnl* with 43.9%/48.4% (gel based/qPCR). Some genogroups considered as rare in the region were detected, such as B (2%) and E (0.66%) by both assays.

## Discussion

Conventional bacteriological methods that are usually routinely used to detect and characterize meningococci are complex, long and involve several overnight cultures and chemical tests which might generate error [15]. Developing a simple and rapid PCR algorithm that detects and characterizes carriage of meningococci is important, especially in the meningitis belt where recent vaccinations with conjugate vaccines were carried out and carriage is usually low [14]. Therefore, it is important to correctly detect *N*. *meningitidis* in a context where transmission is high especially in young children and where the period of carriage is short [34].

In this study, after an *in silico* evaluation of the existing primers and probes, we evaluated a multiplex PCR algorithm by comparison with techniques that were validated and published in a reference laboratory at the University of Oxford (24, 30). Our multiplex PCR algorithm was later used for a meningococcal carriage study in The Gambia and improved the detection of carried meningococci by more than twice (239%) [35].To our knowledge, this is the first time that such an extensive evaluation was made for a carriage study in the meningitis belt, using the most advanced tools and following recommended advanced guidelines for qualitative techniques [32].

To optimise the sensitivity of our algorithm for the detection of meningococci, we targeted both *porA* and *sodC* genes simultaneously. The published *sodC* gel-based PCR primers were re-designed due to too many mismatches against the consensus sequence [18]. Similarly, primers and probe of qPCR targeting *porA* available from the literature [17] yielded too many mismatches and were re-designed entirely. Designing qPCR primers and probes for *cnl* identification was not an easy task as the *cnl* sequence is short and variable, but the design of two complementary forward primers (Table 2), allowed the complete coverage of the *cnl* sequences that were present in pubMLST at that time. Recent comparison of primers/probes sequences used in this study with a larger number of sequences now available in the pubMLST identified additional point mutations in newly published alleles (data not shown). Although most alleles are still covered by the primers and probes developed here, new variations within these primers/probes sequences may lead to decreased sensitivity and we advise a complete new *in silico* evaluation of primers and probes before using these. Since pubMLST is exponentially growing, we recommend our methodology to be applied–or, at a minimum, blasting of primers and probes to make them more robust–when developing or implementing new molecular diagnosis tools for *N*. *meningitidis* as well as other pathogens. The variability of the targeted genes between meningococcal strains might explain the sensitivity and specificity variations observed between monoplex and multiplex assays and future studies will be needed to investigate this further for the detection of *N*. *meningitidis*.

A major strength of the developed algorithm is that the first multiplex assay (*porA*, *sodC* and *cnl*) allows the simultaneous detection of meningococci and the presence/absence of their capsule locus. Since *cnl* genotype is most common among meningococcal strains in carriage [11, 14], this assay improves the rapidity of *N*. *meningitidis* characterization. The sequential use of the following three multiplex assays for genogroup characterization, from the most frequent groups (A, W, and X followed by B, C, and Y) to the carriage uncommon genogroups (E, H and Z) in the meningitis belt, further reduces the time required for meningococcal characterization. The primers/probe sequences used for these genogroup assays could also be modified to fit the variable epidemiology of meningococci by region and over time.

In this study, we have developed an algorithm that: (i) has a high sensitivity for the detection of meningococci; and, (ii) allows rapid characterization of all genogroups with high specificity. A strength of gel-based assays is that the assays joined two commonly used genes to detect *N*. *meningitidis*: *porA* and *sodC* in multiplex, which improved the sensitivity from 70.39% for *porA* and 80.92% for *sodC* alone to 88.82% (see Table 4) for meningococcal detection. However, no improvement in sensitivity and specificity was observed when adding *sodC* in the multiplex qPCR assay and we advise using only *porA/cnl* for the qPCR. This might be due to the modification of the published *sodC* probe to accommodate a MGB quencher in order to harmonize the quenchers in the first multiplex (*porA*, *sodC*, *cnl*). Indeed, “BHQ” probes are difficult to obtain from oligonucleotide suppliers, because of their unusual design [23]. A subsequent comparison of 44 Malian samples tested with both BHQ and MGB probes showed no differences in detection of isolates. However, the efficacy of the shortened MGB *sodC* probe should be further investigated.

For genogroup characterization, multiplex qPCR that has already been evaluated were validated in gel-based PCR platform. The statistical results varied from one genogroup to the other (Table 4). For the two multiplexes (A, W, and X; B, C, and Y), published primers/probes were used and their statistical results were acceptable for all genogroups. The genogroup X primers set had the lowest sensitivity and PPV in the gel-based assay and may require redesign. Overall, the qPCR assay yielded better results than the gel-based PCR and should be the preferred assay for genogroup characterization.

Our study used the laboratory results obtained from Oxford University as reference standard for the statistical analysis. The sensitivity, specificity, PPV and NPV may be affected by the transport conditions when DNA samples were shipped from MenAfriCar countries and how the samples were processed at Oxford University. This may have affected our comparisons between PCR tests realized in Bamako and analyses in Oxford. In addition, no qPCR assay for the rare genogroups H, E, and Z was available in Oxford, making the direct comparison difficult. The results generated in this study were compared to the *ctrA* sequencing results when available and may explain some of the observed discrepancies; indeed, the comparison of two different techniques could explain the low PPV for group E in both assays.

The quality of the template may have also affected the detection of meningococci as most studies use DNA purified by kits. Boiled cell suspensions were purposely used to make our assays as inexpensive as possible and the good statistics obtained with those crude samples demonstrate the efficacy of the assays. Stringent rules were applied to determine whether a test was positive or not: by qPCR, a CT threshold of 30 was chosen based on preliminary results obtained (Fig 2), whereas the CDC/WHO reference guidelines recommend a threshold of 35 which, with the reagents and conditions used in our study, would have led to a considerable number of false positives.

In conclusion, this algorithm allows the detection of *N*. *meningitidis* with high sensitivity for samples from 6 different countries of the meningitis belt, it significantly reduces the number of tests required for meningococcal detection and allows very specific characterization of samples for carriage studies in comparison with conventional bacteriological techniques.

## Supporting information

S1 Fig Primers and probes positioned on appropriate genes. Gel-based (green arrows) and rt (black arrows) PCR primers and probes for each multiplex (A-multiplex 1, B-multiplex 2, C-multiplex 3 and D-multiplex 4); the gene arrow pointing toward the 3’ end of the gene. Both forward primers (available in the literature and newly designed) for *porA* are shown on the gene (A). Figure adapted from Harisson OB and al. 2013.
(PDF)

S1 Table List of papers that were selected for further analyses. (XLSX)

S2 Table Primer and probe sequences general technical analysis. Results of the BLAST analyses performed for each primer and probe using the NCBI BLAST tool. All BLAST results shown were done in the nucleotide collection database, against *N*. *meningitidis* (taxon id 487). The Tm and possible dimers were obtained using the primer express design tool.
(XLSX)

S3 Table A. Statistical results for gel-based PCR assay. Statistical analysis results of the gel-based PCR assay. Sensitivity, Specificity, Positive Predictive Value (PPV) and Negative Predictive Value (NPV) were assessed for each primers pair in the multiplex and monoplex assay pooling results from test on positive controls (n = 16) and the Malian samples (n = 44) and presented as percentages (%). B. Statistical results for the qPCR assay. Statistical analysis results of the qPCR assay. Sensitivity, Specificity, Positive Predictive Value (PPV) and Negative Predictive Value (NPV) were assessed for each primers pair in the multiplex and monoplex assay pooling results from test on positive controls (n = 16) and the Malian samples (n = 44) and presented as percentages (%).
(DOCX)

S1 Dataset Database used for the analyses for the Chadian Site. (XLSX)

S2 Dataset Database used for the analyses for the Ethiopian Site. (XLSX)

S3 Dataset Database used for the analyses for the Ghanaian Site. (XLSX)

S4 Dataset Database used for the analyses for the Malian Site. (XLSX)

S5 Dataset Database used for the analyses for the Niger Site. (XLSX)

S6 Dataset Database used for the analyses for the Senegalese Site. (XLSX)
