# Supplementary figures and images for: Development of a PCR algorithm to detect and characterize Neisseria meningitidis carriage isolates in the African meningitis belt
Source: PLoS One. 2018 Dec 5;13(12):e0206453. doi: 10.1371/journal.pone.0206453 (PMC6281270; doi:10.1371/journal.pone.0206453)

A

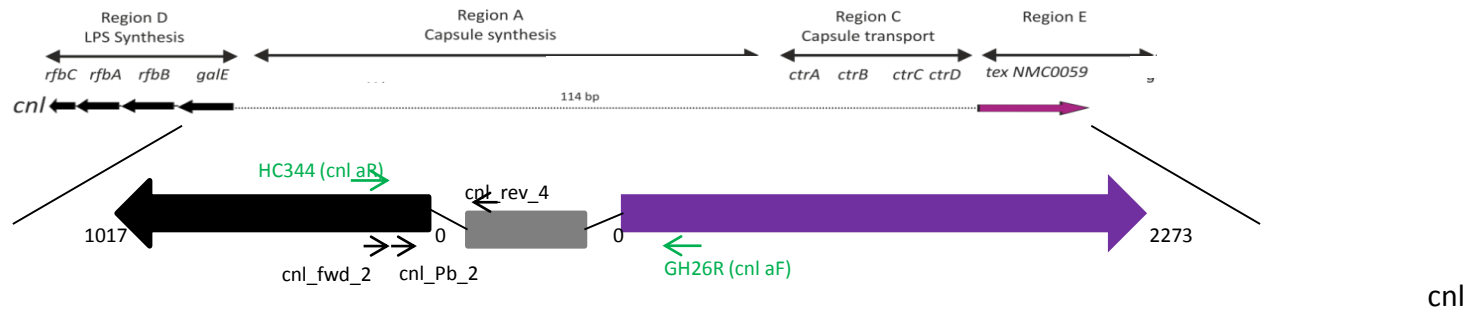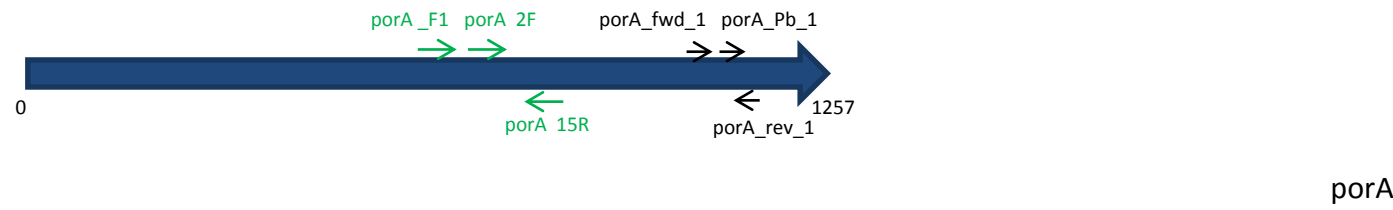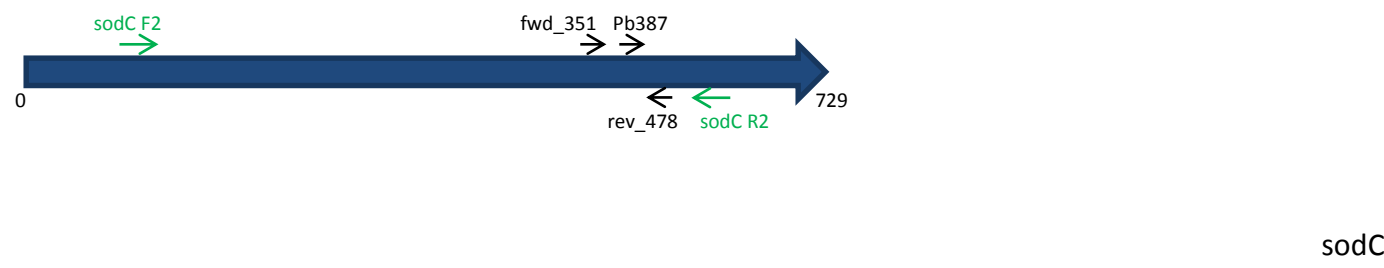

Figure S1 A

B

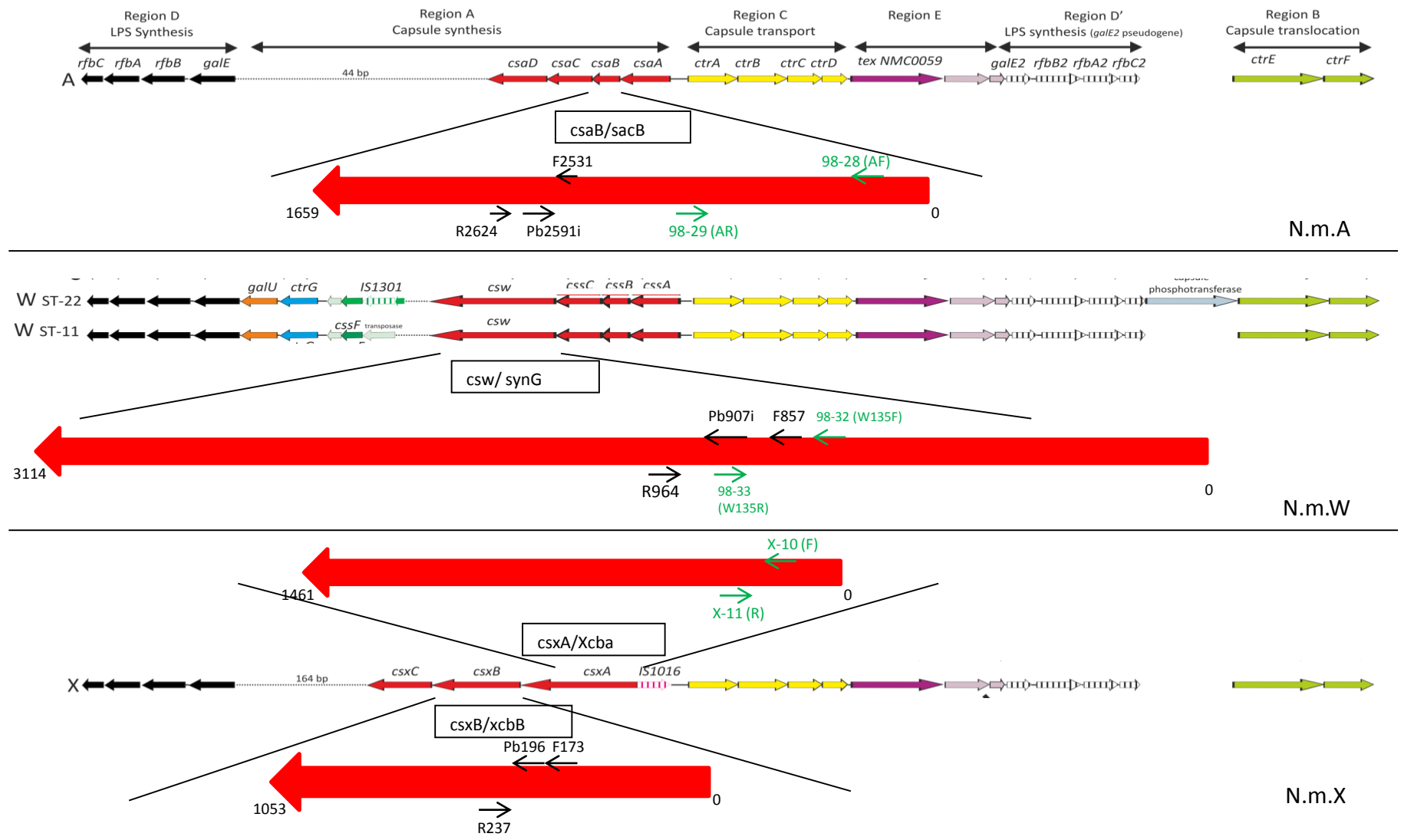

Figure S1 B

C

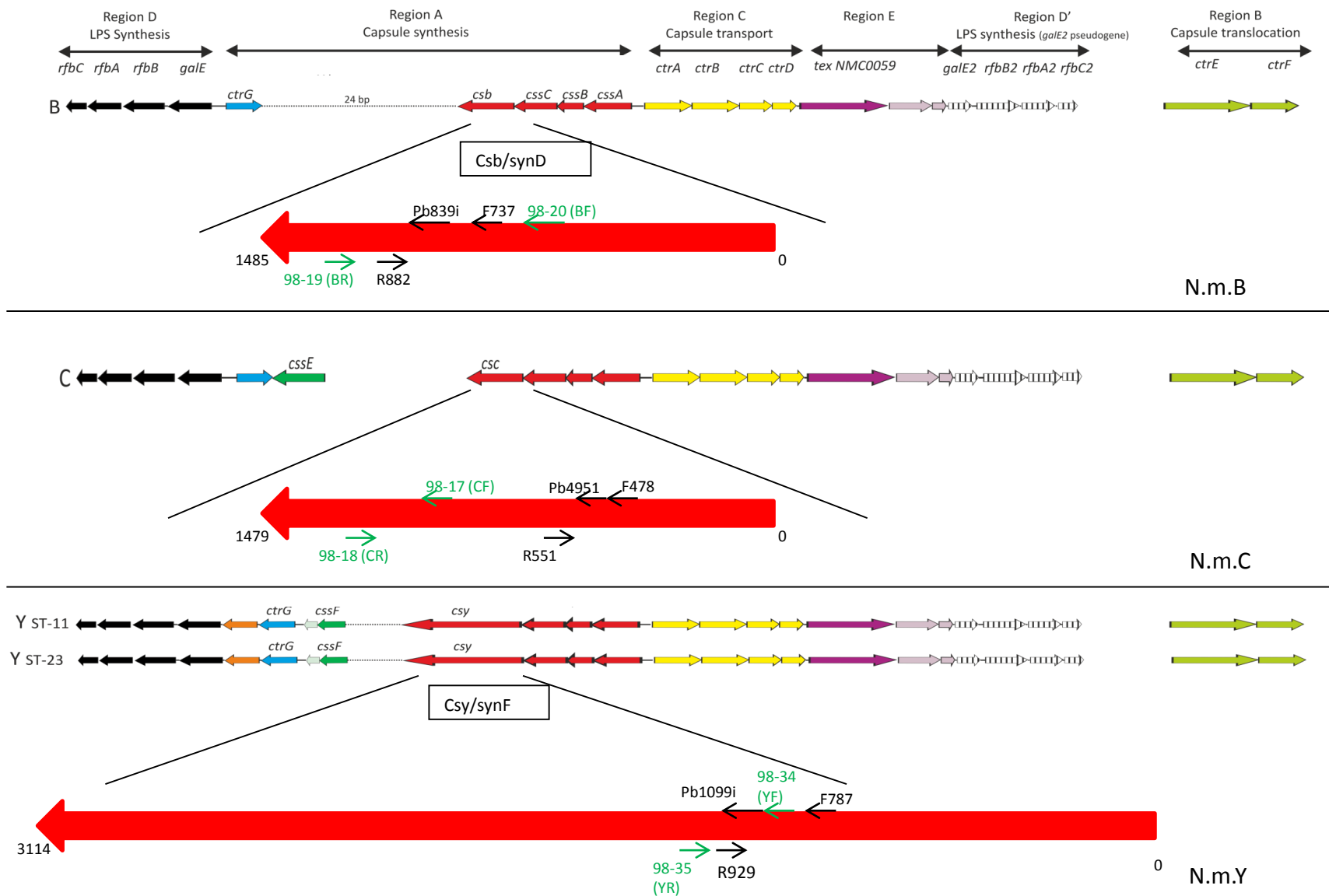

Figure S1 C

D

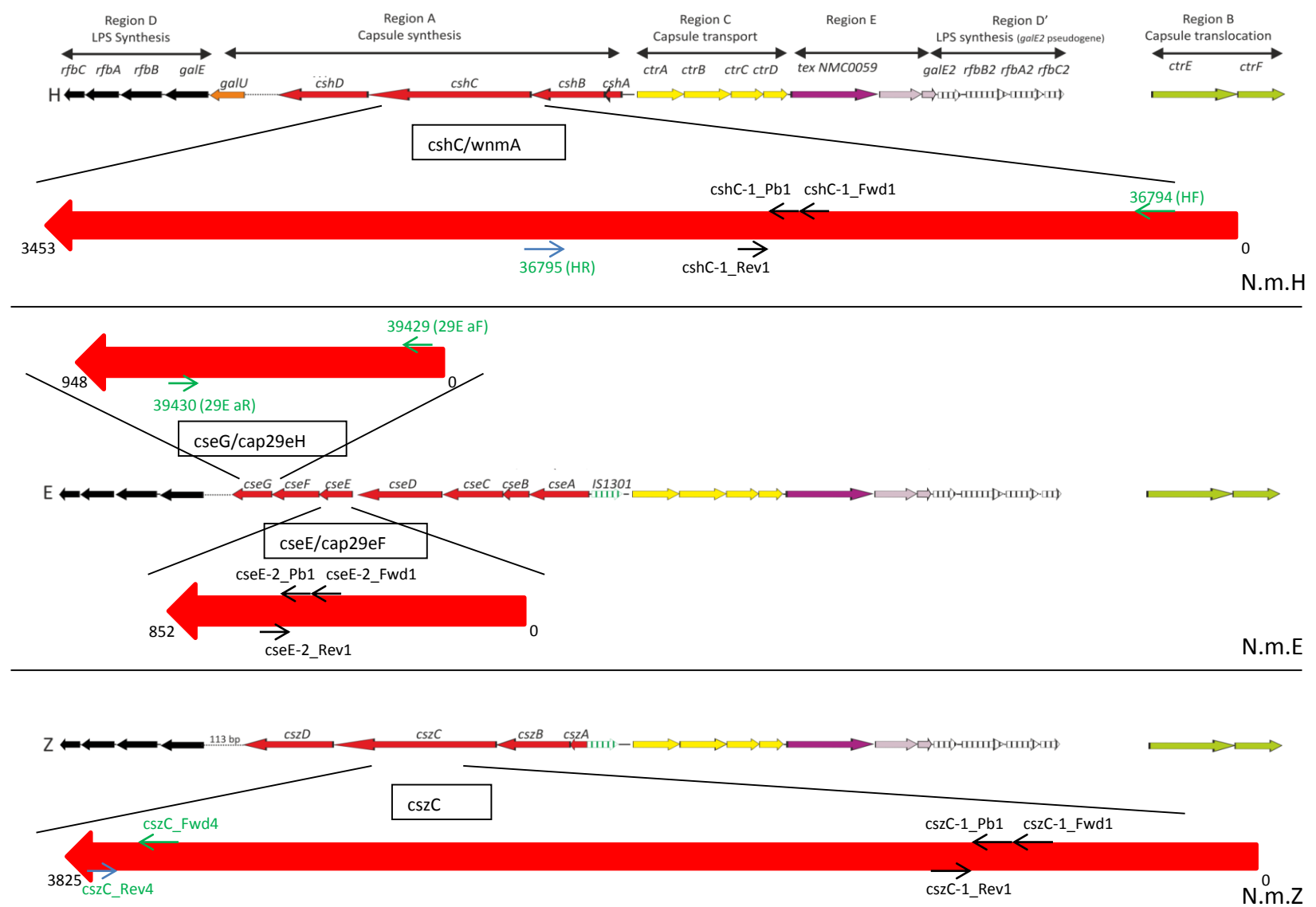

Figure S1 D

Supplement: S1 Fig — Gel-based (green arrows) and rt (black arrows) PCR primers and probes for each multiplex (A-multiplex 1, B-multiplex 2, C-multiplex 3 and D-multiplex 4); the gene arrow pointing toward the 3’ end of the gene. Both forward primers (available in the literature and newly designed) for porA are shown on the gene (A). Figure adapted from Harisson OB and al. 2013. (PDF) [file pone.0206453.s001.pdf]
