# Supplementary material for: Development of a PCR algorithm to detect and characterize Neisseria meningitidis carriage isolates in the African meningitis belt
Source: PLoS One. 2018 Dec 5;13(12):e0206453. doi: 10.1371/journal.pone.0206453 (PMC6281270; doi:10.1371/journal.pone.0206453)
Supplement: S3 Table — A. Statistical results for gel-based PCR assay. Statistical analysis results of the gel-based PCR assay. Sensitivity, Specificity, Positive Predictive Value (PPV) and Negative Predictive Value (NPV) were assessed for each primers pair in the multiplex and monoplex assay pooling results from test on positive controls (n = 16) and the Malian samples (n = 44) and presented as percentages (%). B. Statistical results for the qPCR assay. Statistical analysis results of the qPCR assay. Sensitivity, Specificity, Positive Predictive Value (PPV) and Negative Predictive Value (NPV) were assessed for each primers pair in the multiplex and monoplex assay pooling results from test on positive controls (n = 16) and the Malian samples (n = 44) and presented as percentages (%). (DOCX) [file pone.0206453.s004.docx]

Table A.

| Gel-based | |  | | |  | | |  | | |  |  |  |  |  |  |
| --- | --- | --- | --- | --- | --- | --- | --- | --- | --- | --- | --- | --- | --- | --- | --- | --- |
| monoplex | |  | | |  | | |  | | |  |  |  |  |  |  |
|  | Sensitivity | | Specificity | | | PPV | | | NPV | | |  |  |  |  |  |
| porA | 100,00 | | 77,78 | | | 82,35 | | | 100,00 | | |  |  |  |  |  |
| sodC | 100,00 | | 29,63 | | | 59,57 | | | 100,00 | | |  |  |  |  |  |
| Cnl | 100,00 | | 100,00 | | | 100,00 | | | 100,00 | | |  |  |  |  |  |
| A | 100,00 | | 100,00 | | | 100,00 | | | 100,00 | | |  |  |  |  |  |
| W | 100,00 | | 100,00 | | | 100,00 | | | 100,00 | | |  |  |  |  |  |
| X | 100,00 | | 96,55 | | | 66,67 | | | 100,00 | | |  |  |  |  |  |
| B | 100,00 | | 100,00 | | | 100,00 | | | 100,00 | | |  |  |  |  |  |
| C | 100,00 | | 100,00 | | | 100,00 | | | 100,00 | | |  |  |  |  |  |
| Y | 75,00 | | 100,00 | | | 100,00 | | | 96,15 | | |  |  |  |  |  |
| H | 100,00 | | 100,00 | | | 100,00 | | | 100,00 | | |  |  |  |  |  |
| E | 100,00 | | 100,00 | | | 100,00 | | | 100,00 | | |  |  |  |  |  |
| Z | 100,00 | | 96,30 | | | 50,00 | | | 100,00 | | |  |  |  |  |  |
|  |  |  | |  | | |  | | |  | | |  |  |  |  |
| Gel-based | |  | |  | | |  |  |  |  |  |  |  |  |  |  |
| multiplex |  |  | |  | | |  |  |  |  |  |  |  |  |  |  |
|  | Sensitivity | | Specificity | | | PPV | | | NPV | | |  |  |  |  |  |
| porA | 82,14 | | 88,89 | | | 88,46 | | | 82,76 | | |  |  |  |  |  |
| sodC | 96,43 | | 25,93 | | | 57,45 | | | 87,50 | | |  |  |  |  |  |
| Cnl | 100,00 | | 100,00 | | | 100,00 | | | 100,00 | | |  |  |  |  |  |
| A | 100,00 | | 100,00 | | | 100,00 | | | 100,00 | | |  |  |  |  |  |
| W | 100,00 | | 100,00 | | | 100,00 | | | 100,00 | | |  |  |  |  |  |
| X | 100,00 | | 96,55 | | | 66,67 | | | 100,00 | | |  |  |  |  |  |
| B | 100,00 | | 96,15 | | | 75,00 | | | 100,00 | | |  |  |  |  |  |
| C | 100,00 | | 100,00 | | | 100,00 | | | 100,00 | | |  |  |  |  |  |
| Y | 100,00 | | 100,00 | | | 100,00 | | | 100,00 | | |  |  |  |  |  |
| H | 100,00 | | 100,00 | | | 100,00 | | | 100,00 | | |  |  |  |  |  |
| E | 100,00 | | 100,00 | | | 100,00 | | | 100,00 | | |  |  |  |  |  |
| Z | 100,00 | | 100,00 | | | 100,00 | | | 100,00 | | |  |  |  |  |  |
| porA/sodC | 100,00 | | 25,93 | | | 45,95 | | | 100,00 | | |  |  |  |  |  |

Table B.

| Real-time |  | |  | |  | |  | |  |  |  |  |  |
| --- | --- | --- | --- | --- | --- | --- | --- | --- | --- | --- | --- | --- | --- |
| monoplex | |  |  |  |  |  |  |  |  |  |  |  |  |
|  | Sensitivity | | Specificity | | PPV | | NPV | |  |  |  |  |  |
| porA | 100,00 | | 81,48 | | 84,85 | | 100,00 | |  |  |  |  |  |
| sodC | 100,00 | | 25,93 | | 58,33 | | 100,00 | |  |  |  |  |  |
| Cnl | 100,00 | | 100,00 | | 100,00 | | 100,00 | |  |  |  |  |  |
| A | 100,00 | | 100,00 | | 100,00 | | 100,00 | |  |  |  |  |  |
| W | 100,00 | | 100,00 | | 100,00 | | 100,00 | |  |  |  |  |  |
| X | 100,00 | | 96,55 | | 66,67 | | 100,00 | |  |  |  |  |  |
| B | 100,00 | | 100,00 | | 100,00 | | 100,00 | |  |  |  |  |  |
| C | 100,00 | | 100,00 | | 100,00 | | 100,00 | |  |  |  |  |  |
| Y | 100,00 | | 100,00 | | 100,00 | | 100,00 | |  |  |  |  |  |
| H | 100,00 | | 100,00 | | 100,00 | | 100,00 | |  |  |  |  |  |
| E | 100,00 | | 100,00 | | 100,00 | | 100,00 | |  |  |  |  |  |
| Z | 100,00 | | 100,00 | | 100,00 | | 100,00 | |  |  |  |  |  |
|  |  |  | |  | |  | |  | |  |  |  |  |
| Real-time |  | |  | |  | |  | |  |  |  |  |  |
| multiplex |  | |  | |  | |  | |  |  |  |  |  |
|  | Sensitivity | | Specificity | | PPV | | NPV | |  |  |  |  |  |
| porA | 100,00 | | 74,07 | | 80,00 | | 100,00 | |  |  |  |  |  |
| sodC | 100,00 | | 29,63 | | 59,57 | | 100,00 | |  |  |  |  |  |
| Cnl | 100,00 | | 100,00 | | 100,00 | | 100,00 | |  |  |  |  |  |
| A | 100,00 | | 100,00 | | 100,00 | | 100,00 | |  |  |  |  |  |
| W | 100,00 | | 100,00 | | 100,00 | | 100,00 | |  |  |  |  |  |
| X | 100,00 | | 100,00 | | 100,00 | | 100,00 | |  |  |  |  |  |
| B | 100,00 | | 100,00 | | 100,00 | | 100,00 | |  |  |  |  |  |
| C | 100,00 | | 100,00 | | 100,00 | | 100,00 | |  |  |  |  |  |
| Y | 100,00 | | 100,00 | | 100,00 | | 100,00 | |  |  |  |  |  |
| H | 100,00 | | 100,00 | | 100,00 | | 100,00 | |  |  |  |  |  |
| E | 100,00 | | 100,00 | | 100,00 | | 100,00 | |  |  |  |  |  |
| Z | 100,00 | | 100,00 | | 100,00 | | 100,00 | |  |  |  |  |  |
| porA/sodC | 100,00 | | 29,63 | | 47,22 | | 100,00 | |  |  |  |  |  |
